# Soil Amendment Using Biochar and Application of K-Humate Enhance the Growth, Productivity, and Nutritional Value of Onion (*Allium cepa* L.) under Deficit Irrigation Conditions

**DOI:** 10.3390/plants10122598

**Published:** 2021-11-26

**Authors:** Khaled G. Abdelrasheed, Yasser Mazrou, Alaa El-Dein Omara, Hany S. Osman, Yasser Nehela, Emad M. Hafez, Asmaa M. S. Rady, Diaa Abd El-Moneim, Bassam F. Alowaiesh, Salah M. Gowayed

**Affiliations:** 1 Horticulture Department, Faculty of Agriculture and Natural Resources, Aswan University, Aswan 81528, Egypt; khaledsalim@agr.aswu.edu.eg; 2 Business Administration Department, Community College, King Khalid University, Abha 62529, Saudi Arabia; ymazrou@kku.edu.sa or; 3 Department of Agriculture Economic, Faculty of Agriculture, Tanta University, Tanta 31527, Egypt; 4 Department of Microbiology, Soils, Water and Environment Research Institute, Agricultural Research Center, Giza 12112, Egypt; alaa.omara@yahoo.com; 5 Department of Agricultural Botany, Faculty of Agriculture, Ain Shams University, Hadayek Shubra, Cairo 11241, Egypt; hany_osman1@agr.asu.edu.eg; 6 Department of Agricultural Botany, Faculty of Agriculture, Tanta University, Tanta 31527, Egypt; 7 Citrus Research and Education Center, Department of Plant Pathology, University of Florida, 700 Experiment Station Rd., Lake Alfred, FL 33850, USA; 8 Department of Agronomy, Faculty of Agriculture, Kafrelsheikh University, Kafr El-Sheikh 33516, Egypt; emadhafez2012@agr.kfs.edu.eg; 9 Crop Science Department, Faculty of Agriculture (EL-Shatby), Alexandria University, Alexandria 21545, Egypt; asmaa.mohamed@alexu.edu.eg; 10 Department of Plant Production (Genetic Branch), Faculty of Environmental Agricultural Sciences, Arish University, El-Arish 45511, Egypt; dabdelmoniem@aru.edu.eg; 11 Biology Department, College of Science, Jouf University, Sakaka 72341, Saudi Arabia; bfalawish@ju.edu.sa; 12 Department of Botany, Faculty of Agriculture, Suez Canal University, Ismailia 41522, Egypt; salahgowed@yahoo.com

**Keywords:** water scarcity, deficit irrigation, onion, biochar, soil amendment, potassium-humate, antioxidant, nutritional value

## Abstract

Water scarcity, due to physical shortage or inadequate access, is a major global challenge that severely affects agricultural productivity and sustainability. Deficit irrigation is a promising strategy to overcome water scarcity, particularly in arid and semiarid regions with limited freshwater resources. However, precise application of deficit irrigation requires a better understanding of the plant response to water/drought stress. In the current study, we investigated the potential impacts of biochar-based soil amendment and foliar potassium-humate application (separately or their combination) on the growth, productivity, and nutritional value of onion (*Allium cepa* L.) under deficient irrigation conditions in two separate field trials during the 2018/2019 and 2019/2020 seasons. Our findings showed that deficit irrigation negatively affected onion resilience to drought stress. However, these harmful effects were diminished after soil amendment using biochar, K-humate foliar application, or their combination. Briefly, integrated biochar and K-humate application increased onion growth, boosted the content of the photosynthetic pigments, enhanced the water relations, and increased the yield traits of deficient irrigation onion plants. Moreover, it improved the biochemical response, enhanced the activities of antioxidant enzymes, and enriched the nutrient value of deficiently irrigated onion plants. Collectively, these findings highlight the potential utilization of biochar and K-humate as sustainable eco-friendly strategies to improve onion resilience to deficit irrigation.

## 1. Introduction

Onion (*Allium cepa* L., Family: Amaryllidaceae) is a vegetable crop of high economic importance worldwide [1]. It is a nutrient-dense vegetable, which is high in vitamins, minerals, and antioxidants while being low in calories. Moreover, onion bulbs are good sources of bioactive components, such as polyphenols, flavonoids, and other antioxidants that are important for the human diet and might protect from cancer and cardiovascular disease [1,2]. The total area cultivated in 2019–2020 was 63,000 ha, which produced 2 Mt with a productivity of 35.24 t ha^−1^ [3]. The mean of exports attained 350,000 tons [4]. 

Onion is a shallow-rooted plant that needs carefully scheduled irrigation which consists of determining the amount and timing of irrigation applications to achieve high productivity [5]. As a result of climate change, increment in air temperature and decrement in water resources and soil quality are expected; consequently, water stress and soil salinity will increase [6]. Therefore, a smaller amount of irrigation water than recommended should be applied in crop irrigation programs [7]. Nevertheless, under water stress, the onion reduces its photosynthesis, stomatal conductance, osmotic adjustment, evapotranspiration, and accordingly productivity [6,8]. Water stress is the main restriction for crop yield, particularly in arid and semi-arid zones. Exposing onion crops to water shortage during plant development triggered decreases in length, diameter, and weight of bulbs [9]. Immense decreases in physiological processes (photosynthesis and stomatal conductance), bulb yield, and quality were attained owing to subjecting onions to water stress [6,7]. Once water shortage was imposed, leaf area and bulb growth significantly declined, with a decrease of 19–28% in onion yield [7]. 

In arid and semi-arid zones, irrigated lands exposed to salinization are augmenting annually owing to several causes. Water stress, low-quality irrigation water, high air temperature, and high evaporation rate are examples that might trigger salinity-related problems in these zones [10]. Roughly one-third of the irrigated land is negatively influenced by salinization, which supplies almost 40% of the worldwide food production. Among onion-producing countries, Egypt, suffers from problems of salinity and drainage, influencing between 20 and 35% of their irrigated lands, causing a reduction in crop growth and development. As a consequence of climate changes, water stress and soil salinity along with soil properties should be managed for better crop growth and development [11]. 

Recently, much attention has been given to the development of sustainable agriculture. To alleviate water stress and soil salinity impacts on plants, some new approaches have been applied, including soil amendments and foliar spraying. Biochar has been widely demonstrated as a potential soil amendment to increase soil fertility and crop productivity as it increases soil physico-chemical attributes, plant physio-biochemical properties by improving soil pH [12], cation exchange capacity, water, and nutrient retention capacity in soils, which positively affects plant growth and crop productivity as well as alleviating the harmful impacts of water stress and soil salinity. Biochar is a carbon-rich solid obtained through gasification (fast or slow pyrolysis at various temperatures from 250 to 650 °C), which thermally decomposes biomass or organic materials by heating in the absence or limited presence of oxygen [13]. 

Potassium humate (K-humate) has unique physio-biochemical characteristics and natural properties [14]. Moreover, it has many essential elements that result from the chemical and biological humification of animal and plant matter. Foliar application of K-humate could overwhelm the damaging impacts of water stress and soil salinity [15], positively impact enzyme activities, and enhance plant growth and productivity under abiotic stress. Potassium increases the strength of epidermal cell walls protecting plants versus drought by cuticular transpiration and augments plant tolerance to water stress and soil salinity. Foliar application of potassium enhances the growth and volume/weight development of roots that eventually augment the total area for element absorption [16]. 

Foliar application of potassium positively impacts water use probably due to the increased water withholding via decreasing osmotic potential. Regulation of stomatal conductance is a consequence of potassium inflow and outflow in the stomatal guard cells. Moreover, potassium humate improves the rate of nutrient uptake, plant growth, and productivity as a result of a hormone-like effect, activation of photosynthesis, accelerating cell division, increasing the permeability of plant cell membranes, and improving the plant response to salinity and water stress [17]. Potassium humate can be used as a cheap source of potassium [18]. Furthermore, humic acid is one of the main components of humus [19]. Foliar application of potassium humate had significant impacts on plant growth and improvement of production as well as the quality of wheat plants under abiotic stress [20]. Consequently, the hypothesis tested in the present investigation was designed to assess the potential utilization impacts of biochar as a soil amendment and potassium humate as a foliar application on the growth, yields, physiology, and quality of onion plants along with stimulating the level of some antioxidants that will protect plants against water stress in salt-affected soil.

## 2. Results

### 2.1. Soil Amendment Using Biochar and K-Humate Foliar Application Enhanced Onion Growth

**Figure 1:**
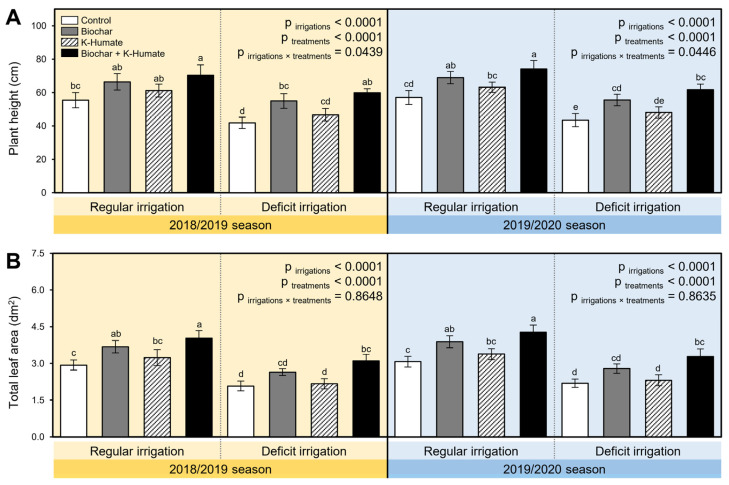
Effect of soil amendment using biochar and K-humate foliar application on the growth of onion plants grown in sodic-saline soil and irrigated regularly (Every 30 days) or stressed with deficit irrigation (every 45 days) during 2018/2019 and 2019/2020 seasons. (**A**) Plant height (cm) and (**B**) Total leaf area (dm^2^). Data presented are the means ± standard deviation (mean ± SD) of three biological replicates. Different letters signify statistically significant differences between treatments according to Tukey’s HSD test (*p*_irrigation×treatment_ ≤ 0.05).

While deficient irrigation significantly reduced the plant height (Figure 1A; *p*_irrigation_ < 0.0001) and total leaf area (*p*_irrigation_ < 0.0001; Figure 1B) in both seasons compared with the regular irrigated onion plants, soil amendment using biochar, K-humate foliar application, or their combination significantly improved both plant height (*p*_treatment_ < 0.0001; Figure 1A) and total leaf area (*p*_treatment_ < 0.0001; Figure 1B). In both seasons, application of biochar individually had a greater effect on both plant height and total leaf area than singular K-humate foliar application. However, combined application of biochar + K-humate had the highest plant height in both growth seasons 2018/2019 (*p*_irrigation×treatment_ = 0.0439) and 2019/2020 (*p*_irrigation×treatment_ = 0.0446) when onion plants were regularly irrigated or stressed with deficient irrigation (Figure 1A). Likewise, biochar + K-humate application increased the total leaf area of onion plants compared to other treatments under both irrigation conditions during the 2018/2019 and 2019/2020 seasons (Figure 1B).

### 2.2. Biochar and K-Humate Application Boosted the Content of the Photosynthetic Pigments of Onion Plants 

**Figure 2:**
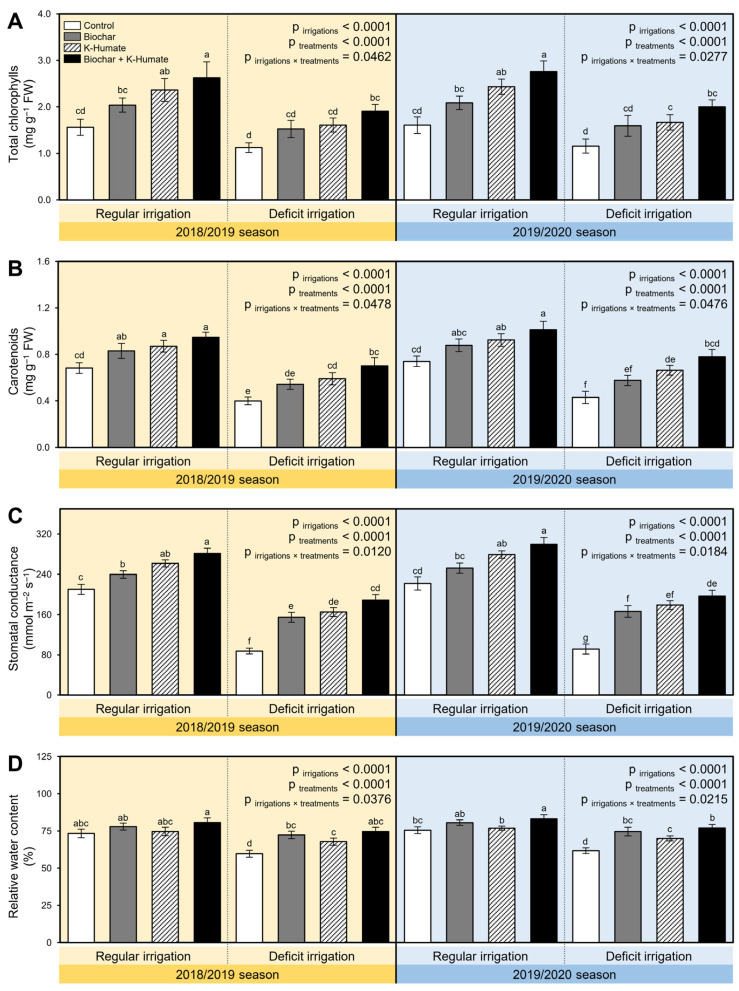
Effect of soil amendment using biochar and K-humate foliar application on the content of the photosynthetic pigments and water relations of onion plants grown in sodic-saline soil and irrigated regularly (every 30 days) or stressed with deficit irrigation (every 45 days) during 2018/2019 and 2019/2020 seasons. (**A**) Total chlorophylls content (mg g^−1^ FW), (**B**) Total carotenoid content (mg g^−1^ FW), (**C**) Stomatal conductance (mmol m^−2^ s^−1^), and (**D**) Relative water content (%). Data presented are the means ± standard deviation (mean ± SD) of three biological replicates. Different letters signify statistically significant differences between treatments according to Tukey’s HSD test (*p*_irrigation×treatment_ ≤ 0.05).

Generally, deficient irrigation significantly decreased the total chlorophylls content (*p*_irrigation_ < 0.0001; Figure 2A) and carotenoid content (*p*_irrigation_ < 0.0001; Figure 2B) of onion plants compared with regularly irrigated plants during the 2018/2019 and 2019/2020 seasons. Nevertheless, these harmful consequences were significantly diminished after soil amendment using biochar, K-humate foliar application, or their combination. Briefly, onion plants treated with biochar + K-humate application had the highest chlorophylls (*p*_irrigation×treatment_ = 0.0462 and 0.0277; Figure 2A) and carotenoid content (*p*_irrigation×treatment_ = 0.0478 and 0.0476; Figure 2B) during the 2018/2019 and 2019/2020 seasons, respectively. It is worth mentioning that individual K-humate foliar applications had a slightly better impact on both chlorophylls and carotenoid content of onion plants than individual biochar application. 

### 2.3. Exogenous Application of Biochar and K-Humate Enhanced Water Relations of Deficiently Irrigated Onion Plants

Water relation attributes including stomatal conductance and relative water content of onion leaves were measured. Even though the deficient irrigation significantly reduced both stomatal conductance (*p*_irrigation_ < 0.0001; Figure 2C) and relative water content (*p*_irrigation_ < 0.0001; Figure 2D) in both seasons, exogenous application of biochar and/or K-humate substantially enhanced both measurements. It is worth mentioning that onion plants grown in normal irrigated soils and treated with biochar + K-humate together had the highest stomatal conductance among all other treatments during the 2018/2019 (*p*_irrigation×treatment_ = 0.0120) and 2019/2020 (*p*_irrigation×treatment_ = 0.0184) seasons (Figure 2C). Similarly, the relative water content of onion plants grown in biochar + K-humate treated soils was higher than other treatments (Figure 2D). 

### 2.4. Soil Amendment Using Biochar and K-Humate Foliar Application Improved the Biochemical Response of Deficiently Irrigated Onion Plants

**Figure 3:**
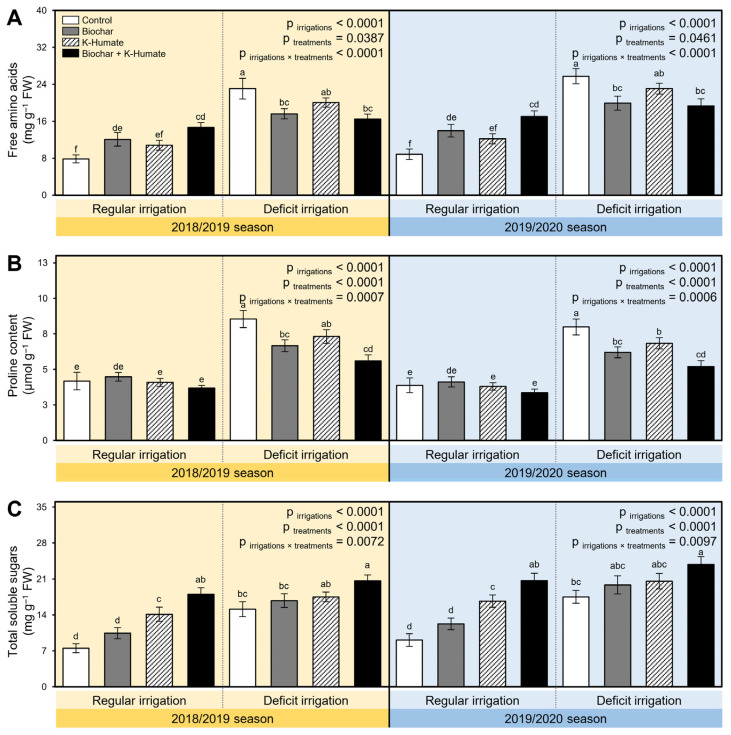
Effect of soil amendment using biochar and K-humate foliar application on the biochemical responses of onion plants grown in sodic-saline soil and irrigated regularly (Every 30 days) or stressed with deficit irrigation (every 45 days) during 2018/2019 and 2019/2020 seasons. (**A**) Free amino acids (mg g^−1^ FW), (**B**) Proline content (µmol g^−1^ FW), and (**C**) Total soluble sugars (mg g^−1^ FW). Data presented are the means ± standard deviation (mean ± SD) of three biological replicates. Different letters signify statistically significant differences between treatments according to Tukey’s HSD test (*p*_irrigation×treatment_ ≤ 0.05).

The influence of soil amendment using biochar and K-humate foliar application on the biochemical response of onion plants, including free amino acids, proline content, and total soluble sugars, were studied in two separate field trials during the 2018/2019 and 2019/2020 seasons (Figure 3). While the irrigation deficiency significantly augmented the three studied biochemical attributes (*p*_irrigation_ < 0.0001) in both seasons, soil amendment using biochar and/or K-humate foliar application significantly increased free amino acids in regularly irrigated onion plants but decreased it in onion leaves under deficient irrigation (*p*_irrigation×treatment_ < 0.0001 in both seasons; Figure 3A). On the other hand, exogenous application of biochar and/or K-humate reduced the proline content in onion plants under both irrigation conditions (*p*_irrigation×treatment_ = 0.0007 and 0.0006; Figure 3B) during 2018/2019 and 2019/2020, respectively. Moreover, the dual application of biochar and K-humate increased the total soluble sugars when onion plants were watered regularly or stressed with deficient irrigation compared with non-treated control (*p*_irrigation×treatment_ = 0.0072 and 0.0097; Figure 3C) in 2018/2019 and 2019/2020, respectively.

### 2.5. Integrated Biochar and K-Humate Application Diminish the Stress Biochemical Indicators in Stressed Onion Plants

**Figure 4:**
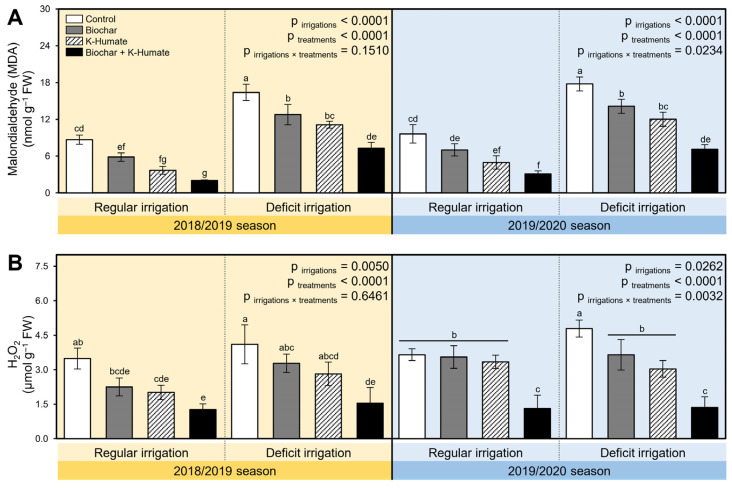
Effect of soil amendment using biochar and K-humate foliar application on the stress biochemical indicators of onion plants grown in sodic-saline soil and irrigated regularly (Every 30 days) or stressed with deficit irrigation (every 45 days) during 2018/2019 and 2019/2020 seasons. (**A**) Malondialdehyde (MDA; nmol g^−1^ FW) and (**B**) H_2_O_2_ (µmol g^−1^ FW). Data presented are the means ± standard deviation (mean ± SD) of three biological replicates. Different letters signify statistically significant differences between treatments according to Tukey’s HSD test (*p*_irrigation×treatment_ ≤ 0.05).

While deficient irrigation significantly increased the stress biochemical indicators as expressed by MDA (*p*_irrigation_ < 0.0001 in both seasons; Figure 4A) and H_2_O_2_ (*p*_irrigation_ = 0.0050 and 0.0262 during 2018/2019 and 2019/2020 seasons, respectively; Figure 4B), integrated soil amendment using biochar and K-humate foliar application significantly reduced the accumulation of both molecules. Briefly, MDA levels were significantly reduced due to the soil amendment using biochar and/or K-humate foliar application in the 2019/2020 season (*p*_irrigation×treatment_ < 0.0234; Figure 4A). Likewise, H2O2 levels were also reduced in both seasons; however, this reduction was only significant in the second season (*p*_irrigation×treatment_ < 0.0032; Figure 4B). It is worth mentioning that there were no significant differences in the H_2_O_2_ levels between the non-treated control and individual application of biochar or K-humate when onion plants were regularly irrigated. Similarly, no significant differences in the H_2_O_2_ levels between biochar-treated or K-humate-treated stressed onion plants.

### 2.6. Integrated Biochar and K-Humate Application Enhanced the Antioxidant-Related Enzymatic Activity in Deficiently Irrigated Onion Plants

**Figure 5:**
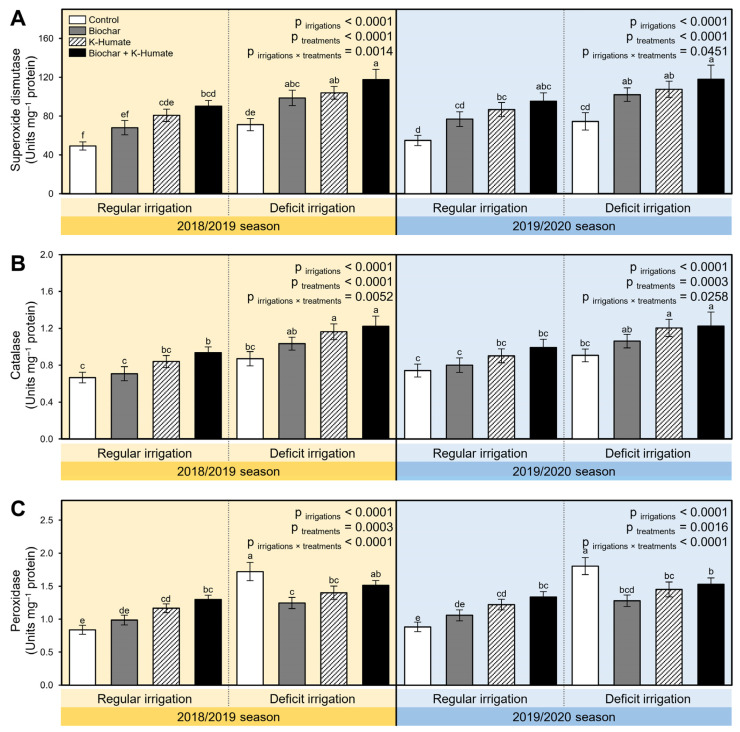
Effect of soil amendment using biochar and K-humate foliar application on the activity of antioxidant-related enzymes of onion plants grown in sodic-saline soil and irrigated regularly (Every 30 days) or stressed with deficit irrigation (every 45 days) during 2018/2019 and 2019/2020 seasons. (**A**) Superoxide dismutase (Units mg^−1^ protein), (**B**) Catalase (Units mg^−1^ protein), and (**C**) Peroxidase (Units mg^−1^ protein). Data presented are the means ± standard deviation (mean ± SD) of three biological replicates. Different letters signify statistically significant differences between treatments according to Tukey’s HSD test (*p*_irrigation×treatment_ ≤ 0.05).

To better understand how soil amendment using biochar and K-humate foliar application alleviate the oxidative stress in deficiently irrigated onion plants, the enzymatic activities of three antioxidant enzymes including superoxide dismutase (SOD; Figure 5A), catalase (CAT; Figure 5B), and peroxidase (POX; Figure 5C) have been colorimetrically determined in two separate field trials during the 2018/2019 and 2019/2020 seasons. Briefly, deficient irrigation significantly increased the enzymatic activities of antioxidant-related enzymes (*p*_irrigation_ < 0.0001; Figure 5). Furthermore, integrated soil amendment using biochar and K-humate foliar application significantly enhanced the activities of all antioxidant enzymes including SOD (*p*_irrigation×treatment_ = 0.0014 and 0.0451; Figure 5A), CAT (*p*_irrigation×treatment_ = 0.0052 and 0.0258; Figure 5B), and POX (*p*_irrigation×treatment_ < 0.0001 and 0.0001; Figure 5C) in the 2018/2019 and 2019/2020 seasons, respectively. It is worth mentioning that although deficient irrigation significantly increased the enzymatic activity of POX in non-treated plants, soil amendment using biochar and/or K-humate foliar application reduced the POX activity in both seasons (Figure 5C).

### 2.7. Soil Amendment Using Biochar and K-Humate Foliar Application Enhanced the Yield Traits of Deficient Irrigated Onion Plants

**Figure 6:**
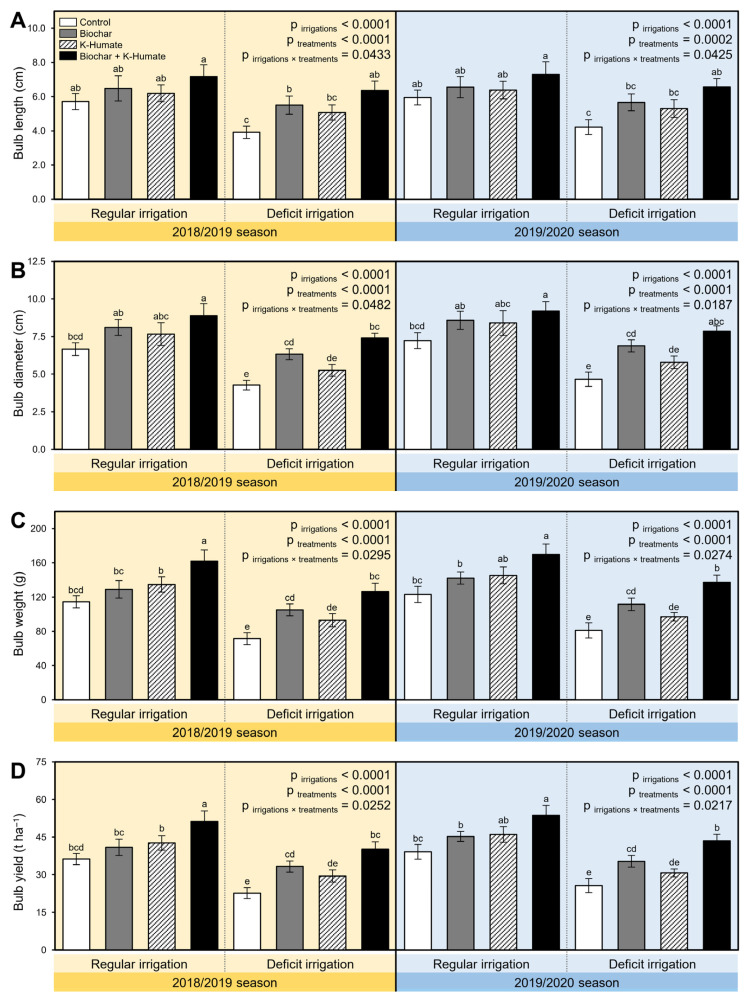
Effect of soil amendment using biochar and K-humate foliar application on the productivity and yield traits of onion plants grown in sodic-saline soil and irrigated regularly (Every 30 days) or stressed with deficit irrigation (every 45 days) during 2018/2019 and 2019/2020 seasons. (**A**) Bulb length (cm), (**B**) Bulb diameter (cm), (**C**) Bulb weight (g), and (**D**) Bulb yield (t ha^−1^). Data presented are the means ± standard deviation (mean ± SD) of three biological replicates. Different letters signify statistically significant differences between treatments according to Tukey’s HSD test (*p*_irrigation×treatment_ ≤ 0.05).

Deficient irrigation negatively altered the yield of stressed onion including bulb length (Figure 6A), bulb diameter (Figure 6B), bulb weight (Figure 6C), and bulb yield (Figure 6D). However, soil amendment using biochar and K-humate foliar application significantly boosted the onion yield. Briefly, the dual application of biochar + K-humate to regularly irrigated soils produced the highest bulb length (7.18 ± 0.69 and 7.30 ± 0.74 cm; Figure 6A), bulb diameter (8.88 ± 0.81 and 9.20 ± 0.62 cm; Figure 6B), bulb weight (161.69 ± 13.37 and 169.70 ± 12.35 g; Figure 6C), and bulb yield (51.18 ± 4.23 and 53.72 ± 3.91 t ha^−1^; Figure 6D) in the 2018/2019 and 2019/2020 seasons, respectively. Likewise, the dual application of biochar + K-humate to deficiently irrigated soils produced the second highest bulb length (6.36 ± 0.55 and 6.57 ± 0.49 cm; Figure 6A), bulb diameter (7.40 ± 0.32 and 7.85 ± 0.37 cm; Figure 6B), bulb weight (126.57 ± 9.61 and 137.48 ± 8.26 g; Figure 6C), and bulb yield (40.06 ± 3.04 and 43.52 ± 2.61t ha^−1^; Figure 6D) during the 2018/2019 and 2019/2020 seasons, respectively. Control onion plants had the lowest bulb length, bulb diameter, bulb weight, and bulb yield during both seasons.

### 2.8. Biochar and K-Humate Supplementation Heightened the N, P, and K Content of Deficient Irrigated Onion Bulbs

**Figure 7:**
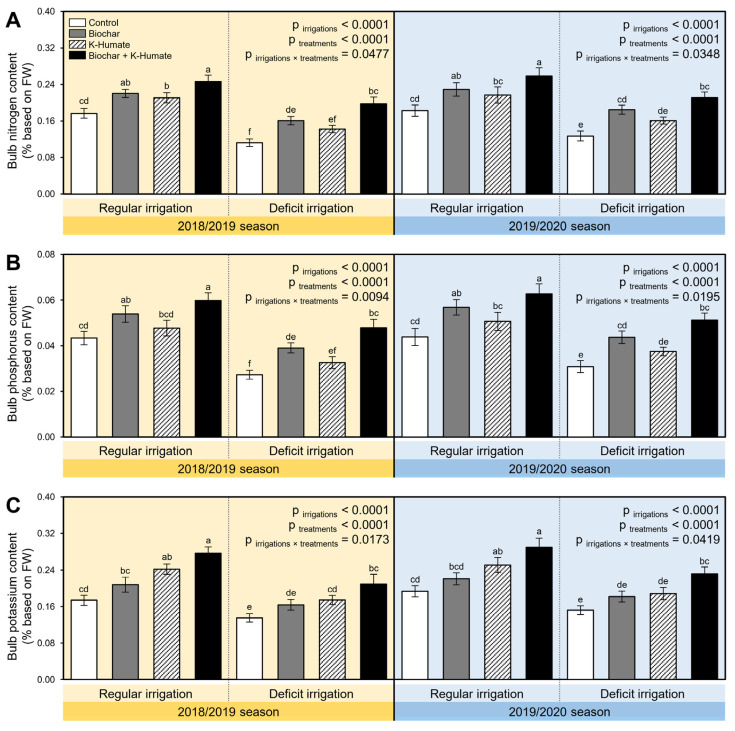
Effect of soil amendment using biochar and K-humate foliar application on the NPK content of onion bulbs grown in sodic-saline soil and irrigated regularly (Every 30 days) or stressed with deficit irrigation (every 45 days) during 2018/2019 and 2019/2020 seasons. (**A**) Bulb nitrogen content (% based on FW), (**B**) Bulb phosphorus content (% based on FW), (**C**) Bulb potassium content (% based on FW). Data presented are the means ± standard deviation (mean ± SD) of three biological replicates. Different letters signify statistically significant differences between treatments according to Tukey’s HSD test (*p*_irrigation×treatment_ ≤ 0.05).

Deficient irrigation significantly reduced the N (*p*_irrigation_ < 0.0001; Figure 7A), P (*p*_irrigation_ < 0.0001; Figure 7B), and K (*p*_irrigation_ < 0.0001; Figure 7C) contents in deficient irrigated onion bulbs during the 2018/2019 and 2019/2020 seasons. However, its undesirable impacts were outstandingly mitigated when onion plants grown in soil was treated with biochar, foliar-treated with K-humate, or their combination (*p*_treatment_ < 0.0001; Figure 7). Under both irrigation conditions, the dual application of biochar and K-humate together had the highest N (*p*_irrigation×treatment_ = 0.0477 and 0.0348; Figure 7A), P (*p*_irrigation×treatment_ = 0.0094 and 0.0195; Figure 7B), and K (*p*_irrigation×treatment_ = 0.0173 and 0.0419; Figure 7C) contents during the 2018/2019 and 2019/2020 seasons, respectively, followed by individual biochar and singular K-humate foliar application during both seasons. However, onion bulbs had the lowest NPK content when grown in non-treated soils under deficient irrigation conditions. 

### 2.9. Combined Biochar and K-Humate Foliar Application Enriched the Nutritional Value of Deficient Irrigated Onion Bulbs

**Figure 8:**
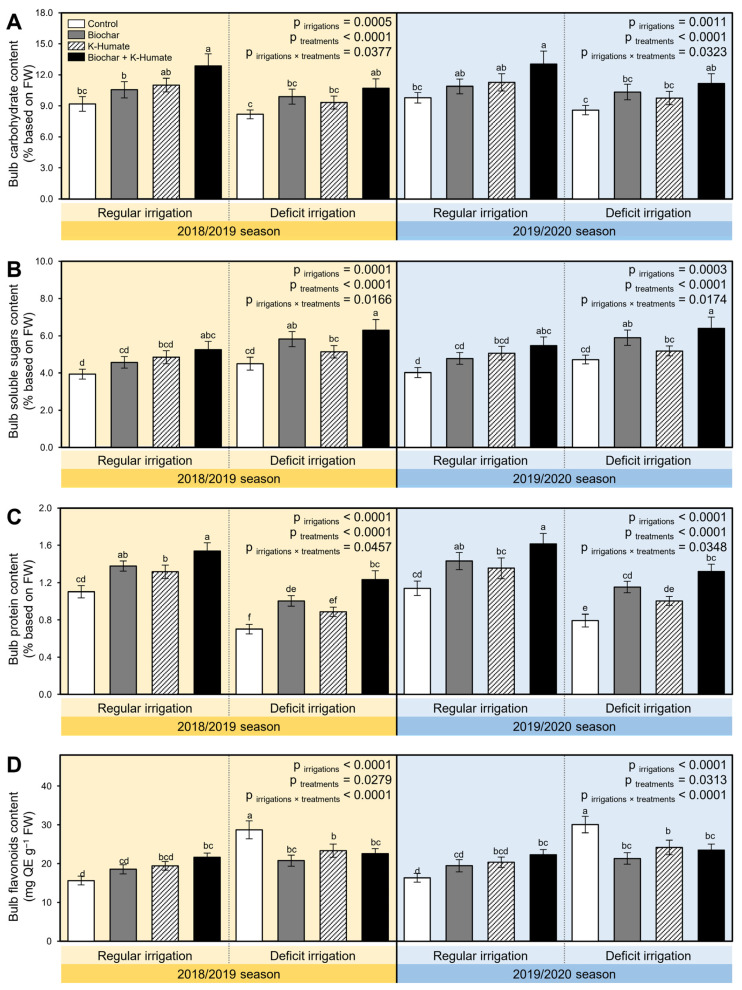
Effect of soil amendment using biochar and K-humate foliar application on the nutritional value of onion bulbs grown in sodic-saline soil and irrigated regularly (Every 30 days) or stressed with deficit irrigation (every 45 days) during 2018/2019 and 2019/2020 seasons. (**A**) Bulb carbohydrate content (% based on FW), (**B**) Bulb soluble sugars content (% based on FW), (**C**) Bulb protein content (% based on FW), and (**D**) Bulb flavonoid content (mg QE g^−1^ FW). Data presented are the means ± standard deviation (mean ± SD) of three biological replicates. Different letters signify statistically significant differences between treatments according to Tukey’s HSD test (*p*_irrigation×treatment_ ≤ 0.05).

Deficient irrigation significantly reduced the nutrient value of stressed onion bulbs, as expressed by carbohydrate (*p*_irrigation_ = 0.0005 and 0.0011 during 2018/2019 and 2019/2020 seasons, respectively; Figure 8A), total soluble sugars (*p*_irrigation_ = 0.0001 and 0.0003 during 2018/2019 and 2019/2020 seasons, respectively; Figure 8B), protein (*p*_irrigation_ < 0.0001 in both seasons; Figure 8C), and flavonoids (*p*_irrigation_ < 0.0001 in both seasons; Figure 8D) contents. 

However, the bulb carbohydrate content (*p*_treatment_ < 0.0001 in both seasons), total soluble sugars content (*p*_treatment_ < 0.0001 in both seasons), protein content (*p*_treatment_ < 0.0001 in both seasons), and flavonoid content (*p*_treatment_ = 0.0279 and 0.0313 during 2018/2019 and 2019/2020 seasons, respectively) were significantly enhanced when onion plants were grown in soil treated with biochar, foliar-treated with K-humate, or their combination. It is worth mentioning that regularly irrigated onion bulbs had the highest carbohydrate content (12.86 ± 1.16 and 13.06 ± 1.24% based on FW) and protein content (1.54 ± 0.09 and 1.61 ± 0.11% based on FW) when they were simultaneously grown in biochar-treated soils and treated with K-humate during the 2018/2019 and 2019/2020 seasons, respectively. Nevertheless, deficient-irrigated onion bulbs had the highest total soluble sugars content (5.25 ± 0.44 and 5.47 ± 0.46% based on FW during 2018/2019 and 2019/2020 seasons, respectively) under combined biochar and K-humate application.

### 2.10. Combined Biochar and K-Humate Application Reduced the Na^+^ Accumulation in Onion Leaves under Deficient Irrigation Conditions

**Figure 9:**
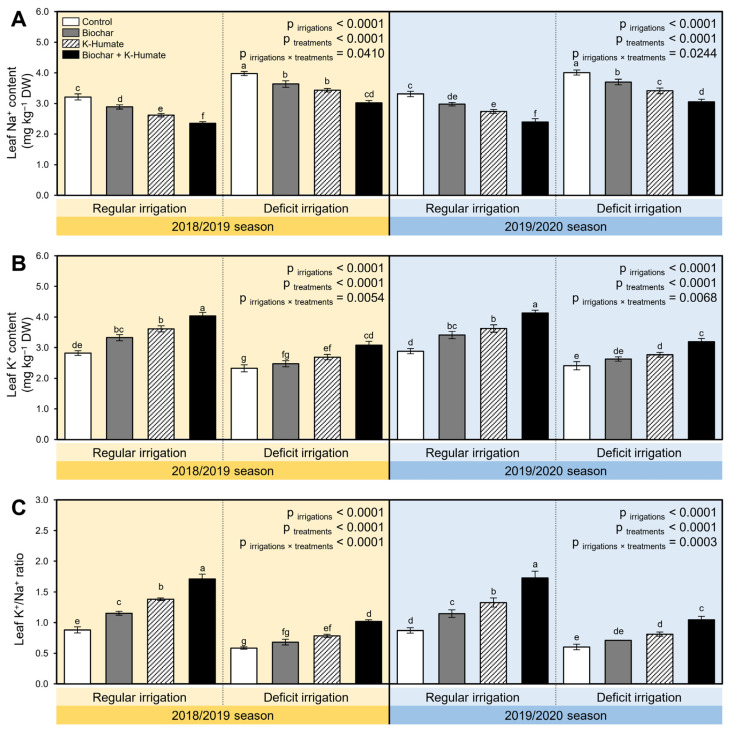
Effect of soil amendment using biochar and K-humate foliar application on the accumulation of Na^+^ and K^+^ in onion leaves grown in sodic-saline soil and irrigated regularly (Every 30 days) or stressed with deficit irrigation (every 45 days) during 2018/2019 and 2019/2020 seasons. (**A**) Leaf Na^+^ content (mg kg^−1^ DW), (**B**) Leaf K^+^ content (mg kg^−1^ DW), and (**C**) Leaf K^+^/Na^+^ ratio. Data presented are the means ± standard deviation (mean ± SD) of three biological replicates. Different letters signify statistically significant differences between treatments according to Tukey’s HSD test (*p*_irrigation×treatment_ ≤ 0.05).

While deficit irrigation conditions significantly increased the accumulation of Na^+^ in onion leaves in both seasons (*p*_irrigation_ < 0.0001, Figure 9A), it notably decreased the K^+^ content in the same leaves (*p*_irrigation_ < 0.0001, Figure 9B). Nevertheless, biochar-based soil amendment and K-humate foliar application substantially reduced the accumulation of Na^+^ but increased the K^+^ content in onion leaves (*p*_treatment_ < 0.0001; Figure 9A,B, respectively). Briefly, while the non-treated onion leaves had the highest Na^+^ content during both seasons, the non-stressed leaves had the highest K^+^ content under both irrigation conditions (Figure 9A,B, respectively). As a result, the K^+^/Na^+^ ratio was notably reduced during both seasons upon the combined biochar and K-humate application (Figure 9C). The lowest K^+^/Na^+^ ratio of all tested treatments was recorded with non-treated stressed plants. However, soil amendment using biochar and/or K-humate foliar application significantly boosted the K^+^/Na^+^ ratios during both seasons (*p*_irrigation×treatment_ < 0.0001 and = 0.0003 during 2018/2019 and 2019/2020 seasons, respectively: Figure 9C).

### 2.11. Principal Component Analysis (PCA) and Two-Way Hierarchical Cluster Analysis (HCA) Revealed the Differences among Treatments

**Figure 10:**
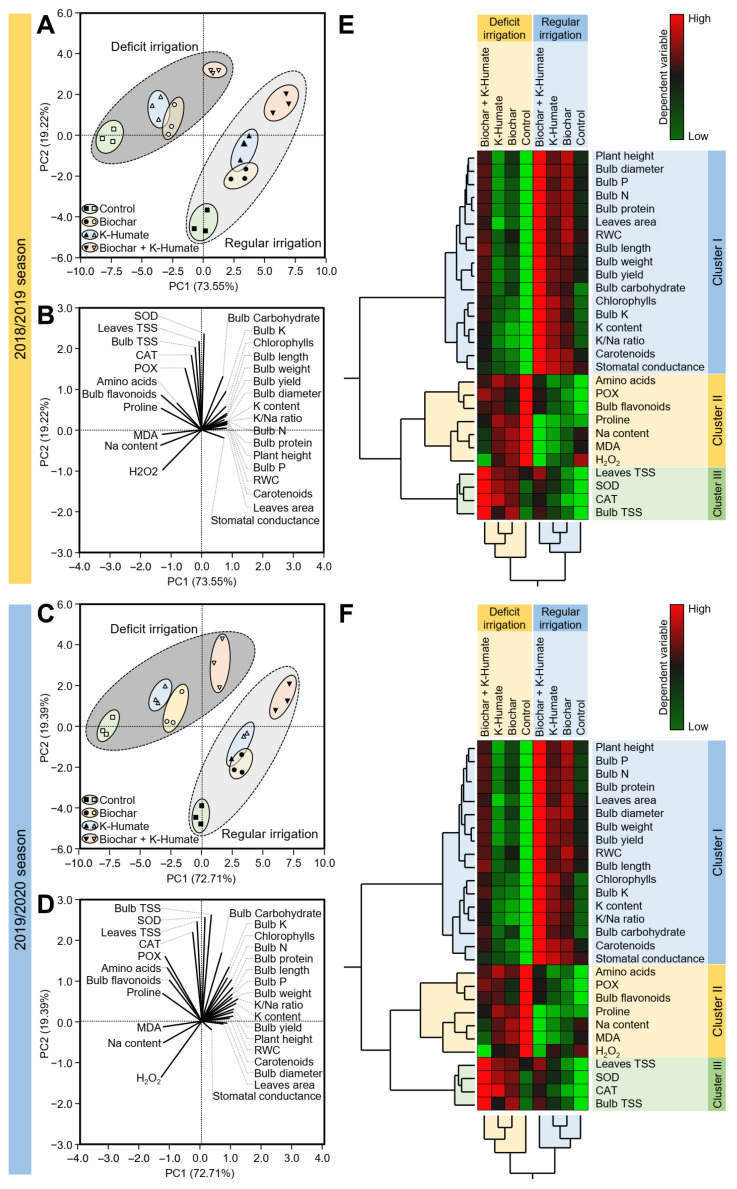
Principal component analysis (PCA) and two-way hierarchical cluster analysis (HCA) of individual response variables assessed in onion plants grown in sodic-saline soil and irrigated regularly (Every 30 days) or stressed with deficit irrigation (every 45 days) after soil amendment using biochar and/or K-humate foliar application during 2018/2019 and 2019/2020 seasons. (**A**,**C**) PCA-associated scatter plots during 2018/2019 and 2019/2020 seasons, respectively, (**B**,**D**) PCA-associated loading plots during 2018/2019 and 2019/2020 seasons, respectively, (**E**,**F**) Two-way HCA during 2018/2019 and 2019/2020 seasons, respectively. Variations in the dependent variables among studied treatments are visualized as a heat map. Rows correspond to dependent variables, whereas columns correspond to different treatments. Low numerical values are green colored, while high numerical values are colored red (see the scale at the top right corner of the heat map).

To better understand our findings, principal component analysis (PCA) and two-way hierarchical cluster analysis (HCA) were carried out (Figure 10). Briefly, the PCA-associated scatter plot showed a clear separation among irrigation strategies (regular irrigation vs. deficient irrigation), as well as all studied treatments (non-treated control, biochar, K-humate, and biochar + K-humate treated) with respect to PC1 (around 73%) and PC2 (approximately 19%) (Figure 10A,C). Additionally, the PCA-associated loading plot showed that while TSS (leaves and bulb), antioxidant-related enzymatic activity (SOD, CAT, and POX), amino acid content, proline content, and bulb flavonoid content were positively correlated with biochar and/or K-humate foliar application, whereas MDA, Na content, and H_2_O_2_ were associated with the non-treated control under deficient irrigation conditions, all other studied variables were positively correlated with the singular biochar application or dual application of biochar and K-humate together under regular irrigation conditions (Figure 10C,D).

In agreement with PCA results, the HCA and its associated heatmap revealed the differences among treatments during the 2018/2019 and 2019/2020 seasons (Figure 10E,F, respectively). Briefly, in both seasons, the HCA-associated dendrogram among treatments showed that all treatments were clustered separately into two distinct clusters (regular irrigation vs. deficient irrigation). Under both irrigation conditions, biochar and K-humate were closer to each other than the dual biochar + K-humate application, whereas the non-treated control was clustered separately under both irrigation conditions during both seasons. Furthermore, the HCA-associated dendrogram among studied variables showed that all examined variables were clearly clustered into three distinct clusters. Cluster ‘I’ included plant height, bulb diameter, bulb NPK content, bulb protein content, leaf area, RWC, bulb length, bulb weight, bulb yield, total chlorophylls content, bulb carbohydrate content, K content, K/Na ratio, total carotenoid content, and stomatal conductance, which all were higher in regularly irrigated onion plants with superiority of biochar + K-humate application (Figure 10E,F). On the other hand, Cluster ‘II’ included free amino acids content, POX, bulb flavonoids, proline content, Na content, MDA, and H_2_O_2_ levels, which all were higher in onions grown under deficient irrigation conditions with a greater effect of the non-treated control. Cluster ‘III’ included leaves TSS, bulb TSS, and enzymatic activity of SOD and CAT, which all were higher in treated onions grown under deficient irrigation conditions, but not the non-treated control.

## 3. Discussion

Application of soil amendment likes biochar or foliar-applied with potassium humate (KH) on plants showed significant impacts on the plant growth, productivity, and quality [21]. Therefore, the application of biochar to salt-affected soil, potassium humate on onion plants, and their combination under deficient irrigation conditions had a positive impact on plant growth and productivity as well as bulb quality traits.

The current research examined an arid zone. Deficient irrigation and salt-affected soil harmfully affected nutrient balance in the soil by cumulative soil exchangeable sodium percentage (ESP). The lessening in plant height and total leaf area under deficient irrigation and salt-affected soil is probably due to the reduction in the transport and translocation of K^+^, Ca^2+^, and Mg^2+^ ions [5]. It was found that the application of biochar could enhance the phenotypic parameters like plant height and total leaf area due to increment of cell division and enlargement [22]. So, the vegetative growth in onion plants confidently responded to biochar addition under deficient irrigation and soil salinity. Biochar had the potential to enhance the nutrients and water absorbance by onion plants and increase the plant growth as expressed by plant height and total leaf area [5].

Additionally, foliar spraying with potassium humate produced a higher vegetative growth such as plant height and total leaf area than control (untreated plots) and lower than biochar application [18]. The enhancement of onion plant growth in response to foliar spraying of potassium humate may be ascribed to its contents of proteins, amino acids, different nutrients, and a higher percentage of vitamin B that can play a crucial role in improving plant growth [23]. It has been reported previously that the foliar spraying of potassium humate augmented plant growth traits on vegetable crops [9]. Furthermore, the increase of onion plants by spraying potassium humate can be ascribed to its contents of auxins (indole-3-acetic acid and indole-3-butyric acid) and cytokinins [24]. Soil and foliar application together by biochar and potassium humate caused a significant increment in phenological characteristics such as plant height and total leaf area compared to the single application or without such application.

Onion plants exposed to water stress under salt-affected soil reduce the plant growth and development by interrupting various physiological characteristics, such as total chlorophyll, carotenoids, stomatal conductance, and relative water content in addition to biochemical properties like total amino acids, proline content, and total soluble sugars [25]. In the current study, it was found that total chlorophylls, carotenoids, stomatal conductance, relative water content, and total soluble sugars were significantly increased and total amino acids and proline content decreased due to the important role of dual application of soil amendment with biochar and foliar spraying with potassium humate which had the potentiality to stimulate the meristematic activity which increases cell division and enlargement [26,27]. 

Application of biochar to the salt-affected soil could increase solubilize inorganic forms, nutrient availability, and uptake which improve soil quality causing enhancement of soil physiochemical properties which reflecting positively on the plant physiological and biochemical characteristics. Similar findings were stated by Hafez et al. [13]. Though foliar application with potassium humate has been shown to enhance physiological and biochemical characteristics like total chlorophyll, carotenoids, stomatal conductance, and relative water content as well as total amino acids, proline content, and total soluble sugars in onion plants under water stress conditions in salt-affected soil thought to be associated with the hormonal substances present in the potassium humate, especially cytokinins [9]. A similar result was reported by Mesbah [28]. The impact of potassium humate may be attributed to its positive contribution to cell respiration, photosynthesis, formation of structural proteins, and other enzymatic activities [10,29]. Moreover, rich nutrients observed in potassium humate such as potassium and trace elements (Fe, Cu, Zn, Co, Mo, Mn, and Ni) may be released through its break-down [14,15]. Potassium humate applied combined with biochar more positively affected physiological and biochemical traits than a sole application under deficient irrigation in soil salinity. It was found that the coupled application significantly increased nutrient availability and membrane permeability, more so than individual applications.

It is an undoubted fact that reactive oxygen species are concerned with cell signaling and membrane potential. ROS are known to be essential participants in the complex signaling network that plants use to respond to abiotic stress like drought and salinity as well as regulate physiological and biochemical parameters. The formation of excess amounts of ROS under abiotic stress led to increasing the damage level in cell compartments due to oxidation effects of free radicals like H_2_O_2_. Lipid peroxidation in the form of MDA formation is considered as one of the best oxidative stress indicators, which give a direct indication of the tolerance level to stress conditions. Hence, the content of H_2_O_2_ and MDA in plant cells has been used to assess plant response to the impact of water stress and soil salinity [30]. Under deficit irrigation, non-treated plants (control plants) produce higher amounts of particular ROS like H_2_O_2_, which is reflected in increasing the level of MDA as an indicator for lipid peroxidation, which constrains plant metabolic activities [31]. However, they were produced in lesser amounts when treated with the combined application of biochar and K-humate. Plant cells usually reduce oxidative damage by synthesizing antioxidants, which are recognized as ROS scavengers [32]. In conformity with our findings, the activity of antioxidant enzymes such as SOD, CAT, and POX in onion leaves was increased under water stress in salt-affected soil. However, the combined application of biochar and K-humate had significant positive impacts. Enzymatic antioxidants of SOD, CAT, and POX reduce H_2_O_2_ levels and MDA that reduce the membrane peroxidation, therefore preserving normal cell functions. Our data are in harmony with the results of Ahmad et al. [33], where biochar reduced the harmful impacts of water stress and soil salinity. It was stated that coupled application of biochar along with K-humate reduced the negative impacts of water stress and soil salinity on onion growth by improving SOD, CAT, and POX activities as well as lowering production of H_2_O_2_ and MDA [34,35].

The antioxidant enzymes, i.e., SOD, CAT, and POX, significantly augmented in onion plants exposed to deficient irrigation under salt-affected soil that positively reflected on the decline of the oxidative stress and scavenged reactive oxygen species [36]. It was obvious from the current study that the application of biochar improved SOD, CAT, and POX activities which maintained membrane integrity and alleviated the harmful effects of drought and salinity-induced oxidative stress [37,38]. Foliar spraying with potassium humate to onion plants increased the enzymatic activity and alleviated the abiotic factors that maintain plant development through encouraging the antioxidant defense system under water stress and salt-affected soil [19]. Foliar spraying with potassium humate to onion plants showed positive effects on enhancing the activity of antioxidant enzymes which regulate the osmotic pressure, protect plant cells from oxidative stress, and improve the electron transport chain [3,20]. Thus, the synergistic application of biochar and potassium humate gave a higher improvement in SOD, CAT, and POX than sole applications. Similar findings were reported previously [17,19].

In the current study, the decline in the bulb length, bulb diameter, and bulb weight that harmfully influenced bulb yield under deficient irrigation and salt-affected soil was attributed to the prevention in the uptake and distribution of the nutrition materials during the growth of bulbs [5]. Moreover, salt-affected soil may introduce severe damage to the bulb and therefore can result in a decline in the bulb yield [39]. With regard to yield and its related traits, the sole application of potassium humate or biochar applications had a beneficial impact in increasing onion yield traits like the bulb length, bulb diameter, and bulb weight as a result of alleviating the injurious effect of deficient irrigation and salt-affected soil relative to control plots which can increment the sterility under abiotic stressors. Application of biochar could improve potassium uptake and decrease sodium uptake, maintaining bulb length, bulb diameter, and bulb weight that positively reflect on photosynthesis and eventually increase bulb yield under water stress in salt-affected soil [36,37]. Moreover, the application of potassium humate increased potassium uptake, decreased osmotic stress and also increased translocation from sources to sink [40]. In addition, starch accumulation in the chloroplast reduced oxidative stress [20]. It was reported that coupled application of biochar and potassium humate that stated high bulb length, bulb diameter, and bulb weight resulted in high bulb yield under water stress and soil salinity.

Herein, deficit irrigation significantly reduced bulb N, P, and K content compared to regular irrigation conditions, leading to a decline of uptake of macro-elements due to lessened transpiration and nutrient absorption in the soil [27]. Reducing nutrient absorption might be ascribed to low soil moisture availability in salt-affected soil that reduces the solubility of nutrients leading to reduction of ions in the soil [13]. The soil application of biochar with foliar spraying of potassium humate to onion plants significantly increased the necessary nutrients in the soil, which boosted nitrification and maintained the water and nitrogenous nutrients in the rhizosphere in comparison with untreated plots [36]. In addition, potassium humate as foliar spraying could sustain higher contents of K in the leaves and inhibit leaf water depletion and augment K absorption. Similar findings were reported previously [20]. The sole addition of biochar could alleviate the harmful impact of deficit irrigation on bulb onion yield by sustaining water holding and osmoregulation, leading to ameliorating soil enzymatic activity and soil physicochemical traits, reflecting positively on bulb N, P, and K content [38]. However, it was observed that the coupled application of biochar with potassium humate further enhanced ion balance, plant physiological traits under low moisture availability under salt stress, leading to augmented bulb yield productivity and nutrient absorption.

In this context, planting onion seeds in soil treated with biochar and/or potassium humate as foliar spraying resulted in great increments in bulb carbohydrate, bulb soluble sugars, bulb protein, and bulb flavonoid contents in comparison with soil without biochar application [5]. The dual application of biochar and K-humate stimulates the stress tolerance of treated plants and improves their adaptation responses to various abiotic stresses [12]. The beneficial effects of biochar and K-humate may be ascribed to the entrance of potassium humate into the cells, carrying both macro-elements and water [3]. Additionally, biochar and/or potassium humate enhance the water permeability through the cell membranes and increase the water holding capacity of treated plants [2]. Potassium humate application enhanced the biosynthesis of organic compounds and this could be directly linked to its role in augmenting the efficiency of plant resistance [27]. This is owing to the boost in metabolism by enhancing photosynthesis, nutrient uptake, and translocation inside the plant and its metabolism, thus increasing the yield in general [9]. Nutritional contents of onion were enhanced in potassium humate applied as the presence of biochar could have resulted in improved phenolic compound synthesis [28] which has to been linked to a positive relation between bulb carbohydrate, bulb soluble sugars, bulb protein, bulb flavonoid contents and potassium humate applied on onion. Bulb carbohydrate, bulb soluble sugars, bulb protein, and bulb flavonoid contents rely upon transported ions and organic solutes that are converted into glucose inside onion [17,25] where biochar and potassium humate application enhances glucose biosynthesis, contributing to improved quality traits in onion plants.

## 4. Materials and Methods

### 4.1. Experimental Site, Soil, and Meteorological Conditions

Two field experiments were performed at the Elamaar village in the region of Sidi Salem (31°07′ N, 30°57′ E), Kafr El-sheik Governorate, Egypt, during two consecutive growing seasons (2018/2019 and 2019/2020) in order to determine the impact of soil application with biochar and foliar application of potassium humate (K-humate; Egyptian Company for Fertilizers and Chemicals, Attaka, Suez Governorate, Egypt) under deficient irrigation on growth, physiological traits, antioxidant activities, productivity, and the nutritional quality of onion plants in salt-affected soils. Soil samples were analyzed at depth 0−20 cm before plantation. Salinity was determined in the saturated soil paste extract according to Klute and Page [41]. Soil bulk density and total porosity were as described by Campbell and Trout et al. [42,43]. Organic matter content was determined according to the Walkaly and Black method as described by Hesse [44]. To investigate the texture of the soil, the particle size distribution was evaluated according to the method of Gee and Bauder [45]. Physiochemical analysis in both seasons is shown in Table 1, whereas meteorological data of the experimental site is shown in Table 2.

### 4.2. Plant Materials and Growth Conditions

Onion (*Allium cepa* L.) cultivar Giza 20 was used as an experimental model throughout this study. Onion seeds were sown at the nursery on the 10th and 17th of October in the 2018/2019 and 2019/2020 seasons, respectively. Onion seedlings were transplanted on December 10th and 19th in the first and second seasons, respectively. Seedling transplantations were at two sides of every ridge and the distance between seedlings was 10 cm. The harvesting was done after 150 days from the transplanting date in both seasons of this study. The two water irrigation regimes were as follows: The recommended irrigation was five irrigations (irrigation every 30 days) which is named regular irrigation and deficient irrigation was three irrigations (irrigation every 45 days); each irrigation treatment was stopped before one month from harvesting; the four amendment treatments included non-treated control, without the application of biochar or K-humate, soil application of biochar, foliar application of K-humate and combined application of biochar and potassium humate. Cultivation practices such as killing weeds, pest, and diseases control were done following the guidelines given by the Ministry of Agriculture. Before onion sowing, ploughing was performed at 40 cm depth and hoeing at 15 cm during soil preparation, the experimental soil was fertilized with phosphorus fertilizer in the form of calcium superphosphate (15.5% P_2_O_5_) at the rate of 107 kg P_2_O_5_ ha^−1^ during land preparation and potassium (K) at the rate of 120 kg ha^−^^1^ in the form of potassium sulfate (48% K_2_O) during seedbed preparation before ridging, and the nitrogen application rate was 286 kg ha^−8^ as ammonium nitrate (33.5%). The nitrogen fertilizer was divided into two parts and half was applied before the second irrigation and the other half before the third irrigation.

### 4.3. Biochar Application

Biochar was made by gradual pyrolysis of rice husk and maize stalk (1:1) at 350 °C in an oxygen-depleted environment for 3 h. Briefly, rice husks and corn stalks were mixed homogeneously and then subjected to slow pyrolysis to produce biochar.

Physico-chemical properties of used biochar were described in our previous studies [21,46]. After air drying, biochar was ground to a fine powder in a stainless-steel mill to eliminate big particles and raked for leveling. Biochar was used at the rate of 1 kg m^−2^ (10 ton ha^−1^) as recommended in previous literature [21,46,47]. Biochar was distributed homogeneously to each plot and mixed diffusely with surface soil (0–20 cm depth) one week prior to transplanting and during the tillage process. Neither the control treatment nor individual K-humate treatment received biochar. 

### 4.4. Potassium-Humate Application

Potassium humate (humic acid: 85%, K_2_O: 8%, and fulvic acid: 3%) was provided by the Egyptian Company for Fertilizers and Chemicals, Attaka, Suez Governorate, Egypt. The foliar solution volume was 450 L ha^−1^ as recommended by the manufacture with slight modification according to Shafeek et al. [48] and Mohsen et al. [49]. K-humate was sprayed by hand sprayer (for experimental plots) until saturation point. Potassium humate (5 g L^−1^) was foliarly applied three times at 40, 60, and 80 days post-transplanting (dpt) of onion seedlings. The application time was early morning or in the evening when the moisture is high in the plant. Additionally, tap water was used as a control treatment

### 4.5. Growth and Morphological Traits

#### 4.5.1. Plant Height

At harvest time (approximately at 150 dpt), ten biological replicates per treatment were collected randomly to measure plant height (cm) from the base of the swelling sheath to the top of the longest tubular blades. 

#### 4.5.2. Total Leaf Area

Total leaf area per plant was measured using the leaf’s area–weight relationship as described by Wallace and Munger [50]. Briefly, at 125 dpt, ten biological replicates per treatment were randomly collected, washed with running water, and then washed again with double-distilled water. Subsequently, 20 leaf discs (1 cm^2^ each) were dried using an oven at 85 °C for 24 h to obtain the discs’ dry weight (DDW). The total leaf area per plant was calculated using Equation (1) as follows:

(1) $$\mathrm{Total}\;\mathrm{leaf}\;\mathrm{area}\;\mathrm{per}\;\mathrm{plant}\;=\;\frac{\mathrm{LDW}}{\mathrm{DDW}}\times \mathrm{DA}$$

where LDW is the total leaf dry weight (g). 

### 4.6. Photosynthetic Pigment Analysis

Assessments of total chlorophylls and carotenoid content were performed at 125 days after transferring in the 5th fully expanded leaf. Chlorophylls and carotenoid content were assessed in 80% acetone extract. After centrifugation (8000 rpm, 20 min) the absorbance was read spectrophotometrically at 663, 646, and 470 nm. The concentrations were computed as given by Lichtenthaler [51]. Equations (2) and (3) were used for calculation as follows:

(2) $$\mathrm{Total}\;\mathrm{Chlorophylls}\;\left(\mathrm{mg}\;g^{-1}\right)=\frac{\left[20.2\left(A_{646}\right)\;+\;8.02\left(A_{663}\right)\right]\;\times \;\mathrm{Vol}.\;\mathrm{of}\;\mathrm{Etract}}{\mathrm{Sample}\;\mathrm{weight}\;\times \;1000}$$

(3) $$\mathrm{Carotenoids}\;\left(\mathrm{mg}\;g^{-1}\right)=\frac{\left[1000\left(A_{470}\right)-3.27\left(12.21\;A_{663}\;-\;2.81\;A_{646}\right)-104\left(20.13\;A_{646}-5.03\;A_{663}\right)\right]\;\times \;\mathrm{Vol}.\;\mathrm{of}\;\mathrm{Extract}}{\mathrm{Sample}\;\mathrm{weight}\;\times \;227\;\times \;1000}$$

### 4.7. Water Relation Analysis

#### 4.7.1. Stomatal Conductance (gs; mmol H_2_O m^−2^ s^−1^)

Stomatal conductance was determined by a dynamic diffusion porometer (Delta-T AP4, Delta-T Devices Ltd., Cambridge, UK) between 9:00 and 11:00 a.m., from the abaxial and adaxial surfaces of three leaves. 

#### 4.7.2. Relative Water Content

Ten biological replicates were randomly chosen from each treatment to measure fresh weight (FW), then soaked in distilled water at 4 °C in darkness for 24 h to measure the turgid weight (TW) [52]. After that, they were oven dried for 24 h at 80 °C to measure dry weight (DW). Relative water content (RWC) was calculated using Equation (4) as follows:

(4) $$\mathrm{Relative}\;\mathrm{water}\;\mathrm{content}\;\left(\mathrm{RWC},\%\right)=\frac{\left(\mathrm{FW}-\mathrm{DW}\right)}{\left(\mathrm{TW}-\mathrm{DW}\right)}\;\times \;100$$

### 4.8. Biochemical Analysis of Onion Leaves 

#### 4.8.1. Free Amino Acids 

Free amino acids were estimated by a high-performance liquid chromatographic (HPLC) system (Shimadzu Corporation, Kyoto, Japan) consisting of a system controller (SCL-6B), an auto-injector (SIL-6B), HPLC pumps (LC-6A), column oven (CTO-6A), fluorescence detector (RF-551), and a Supelcosil LC-18 column (5 mm packing, 150 × 4.6 mm). Briefly, 100 mg of sliced biological replicates was chosen randomly from each plot and dried to a constant weight for 16 h at 80 °C. The extraction was measured to determine free amino acids according to the method of Hansen [53]

#### 4.8.2. Proline Content

The proline content was determined according to the method of Bates et al. [54] with slight modification. Briefly, ten biological replicates were randomly selected from each plot. For each biological replicate, approximately 300 mg of ground leaf tissue was mixed in 10 mL of 3% (*w*/*v*) sulfosalicylic acid in 80 mL distilled water and sieved. Subsequently, 2 mL of the filtrate was mixed with 2 mL of ninhydrin; then 2 mL of glacial acetic acid was added to the mixture, which was heated for 60 min. Proline content was colorimetrically determined by measuring the absorbance of toluene fraction, which aspired from the liquid phase, at 520 nm. Proline content was described as μmol proline g^−1^ fresh weight.

#### 4.8.3. Total Soluble Sugars (TSS)

Leaf samples (0.5 g) were extracted with 80% hot ethanol. The concentration of TSS was estimated based on the anthrone method by Sadasivam and Manickam [55]. Anthrone reagent was prepared by dissolving 200 mg anthrone with 100 mL of 95% H_2_SO_4_. The reaction mixture consists of 100 μL of leaf extract and 2.9 mL of anthrone reagent, which incubated for ten minutes at 100 °C to start the reaction, followed by chilling in an ice bath to stop the reaction. The absorbance was read at 625 nm. The concentration of TSS was determined using the glucose standard curve and expressed as mg g^−1^ FW.

### 4.9. Antioxidant Enzyme Activity and Stress Indicators in Onion Leaves

#### 4.9.1. Antioxidant Enzyme Activity

Samples of onion leaves (1 g each) were ground and homogenized in 5 mL of cold phosphate buffer (50 mM phosphate buffer pH 7.0, containing 1 mM EDTA, 1 mM phenylmethylsulfonyl fluoride, and 1% polyvinylpolypirrolidone) to use as an enzyme extract. The homogenized solutions were centrifuged for 25 min at 10,000× *g* at 4 °C to use the supernatant as enzyme extract. The activity of superoxide dismutase (SOD: 1.15.1.1) was determined based on nitro-blue tetrazolium (NBT) photochemical assay at 560 nm as described by Beauchamp and Fridovich [56]. The activity of catalase (CAT: 1.11.1.6) was performed respecting the reaction between 50 µL enzyme extract and 12.5 mm H_2_O_2_ in the presence of 50 mM K-phosphate buffer (pH 7.0). The reaction started by adding H_2_O_2_ and the absorbance was monitored at 240 nm for 60 s [57]. Peroxidase (POX: 1.11.1.7) activity was determined using o-phenylenediamine as a chromogenic indicator in the presence of H_2_O_2_ and enzyme extract at 417 nm as described by Vetter et al. [58]. The activity of all enzymes is presented as the unit mg^−1^ protein.

#### 4.9.2. Stress Indicators (Lipid Peroxidation (MDA) and H_2_O_2_)

Onion fresh leaf samples were weighed and immediately frozen and stored in liquid nitrogen for further analysis. MDA contents as an indicator for the lipid peroxidation in plant cells were determined based on the method illustrated by Du and Bramlage [59]. Frozen leaf samples (0.5 g) were ground in liquid nitrogen using a mortar and pestle and homogenized in 5 mL 0.1% trichloroacetic acid (TCA) then centrifuged at 10,000× *g* for 15 min. A measure of 1 mL of supernatant was mixed with 4.0 mL of 0.5 % thiobarbituric acid (TBA) in a 20 % TCA solution. The mixture was boiled for 30 min and then quickly cooled in an ice bath. Then it was centrifuged again and the absorbance of the supernatant was measured spectrophotometrically at 532 and 600 nm. The absorption coefficient of MDA (157 mmol^−1^ cm^−1^) was used in the calculation of MDA content, which is expressed as nmol g^−1^ FW.

Hydrogen peroxide was determined following the method of Velikova et al. [60]. The estimation of H_2_O_2_ content was carried out using 0.5 g frozen leaf samples in liquid-N_2_, which was homogenized with 5.0 mL of TCA (0.1%, *w*/*v*). The supernatant was centrifuged at 10,000× *g* for 15 min. Then, the reaction was started by mixing 0.5 mL supernatant with 0.5 mL of 10 mM K-phosphate buffer (pH 7.0) and 1 mL of 1 M KI, and the absorbance was read spectrophotometrically at 390 nm. H_2_O_2_ content was calculated using the standard curve of H_2_O_2_ and presented as µmol g^−1^ FW.

### 4.10. Yield Traits

At maturity time, samples of ten onions were collected randomly from the inner rows of every experimental unit to estimate bulb length and bulb diameter (cm) which was measured by a caliper at the maximum swollen part of the bulb. All the remaining bulbs in each plot were translocated to a shady place on the same day for curing then uprooted; the bulb yields of onion are expressed as average bulb weight (g) and total bulbs yield (t ha^−1^).

### 4.11. Nutritional Value Analyses of Onion Bulbs

#### 4.11.1. N, P, and K Content

At maturity time, onion bulb samples from each experimental plot were collected, weighed, and finely ground, and wet digested with sulfuric acid and perchloric acid (3:1) for elemental analysis as dry weight where nitrogen (N), phosphorus (P), and potassium (K) were elements in the fresh weight (FW) of bulb tissue. The N content was determined by the Kjeldahl method as described by AOAC [61], while P content was calorimetrically measured according to Sparks et al. [62]. The K content was assessed by Atomic Absorption Spectrophotometer (AAS, Perkin Elmer 3300) with a detection limit of 100 ppb [63].

#### 4.11.2. Total Carbohydrate, TSS, Protein, and Flavonoid Content

After harvesting, three biological replicates (bulbs including the shattered outer shells) were collected, washed tightly, and the broken external husks were erased, and scratched with a plastic knife into tiny slices. An aliquot of fresh slices was used to assess the total carbohydrate, TSS, protein, and flavonoid content. Total carbohydrates were determined in the aqueous extract according to Dubois et al. [64]. Reading of total soluble sugars (TSS%) was taken using a hand refractometer calibrated as percent sucrose according to AOAC [61]. Protein content in bulbs was calculated by multiplying nitrogen content by 6.25 [65]. Bulb flavonoid content was assessed by aluminum chloride colorimetric assay according to Zou et al. [66].

### 4.12. Na^+^ and K^+^ Accumulation in Onion Leaves

At 90 dpt, three biological replicates were collected randomly from each plot to determine the accumulation of sodium (Na^+^; mg kg^−1^ DW), potassium (K^+^; mg kg^−1^ DW), and K^+^/Na^+^ ratio in onion leaves. After washing and drying the leaves, they were ashed for six hours at 500 °C in a furnace. Na^+^ and K^+^ concentrations were assessed using AAS (AAS 3300, PerkinElmer LAS (UK) Ltd., Beaconsfield, Bucks HP9 2FX, UK) as described by Sparks et al. [62].

### 4.13. Experimental Design and Statistical Analysis

The field experiments were laid out in a split-plot design with three replications comprising of eight treatment combinations, i.e., two water irrigation regimes (regular irrigation vs. deficit irrigation) in the main plots and four amendment treatments (control, biochar, K-humate, and biochar + K-humate) in subplots. The area of each subplot was 10.5 m^2^, with six ridges (3.5 m long and 3 m wide). All experiments were repeated twice in two successive seasons (2018/2019 and 2019/2020), with at least three biological replicates for each treatment. The analysis of variance (ANOVA) was used to test the significant differences among irrigation regimes (*p*_irrigation_), treatments (*p*_treatment_), and their interaction (*p*_irrigation×treatment_). Tukey’s honestly significant difference (HSD) test was used for post-hoc analysis based on the *p*-value of the interaction between irrigation regimes and treatments (*p*_irrigation×treatment_ < 0.05). ANOVA and Tukey’s test were carried out using JMP Data analysis software, Version 15 [67].

## 5. Conclusions

Our results suggest that the dual application of biochar-based soil amendment and foliar K-humate application can be an efficient, sustainable, and eco-friendly strategy to improve onion resilience to deficient irrigation, particularly in arid and semiarid regions as it substantially reduced the harmful impacts of deficient irrigation in sodic-saline soils. The application of biochar + K-humate enhanced onion growth, which was accompanied by enhanced photosynthetic pigment content and, consequently, the photosynthesis process and finally onion productivity. However, the capability of biochar and K-humate application depends on the biochar source and material types used, the pyrolysis of biochar as well as the soil system. The mechanism by which biochar and K-humate alleviate the negative effects of salinity and deficient irrigation on both plant and soil ecosystems is complex. The economic benefit, as well as the long-term effects of biochar and K-humate and their application rate, should be intensively studied. Therefore, long-term field trials are vitally required to evaluate the impact of biochar and K-humate application on plants grown in sodic-saline soils.

## Figures and Tables

**Table 1:** Soil properties of the experimental sites (soil depth 0–20 cm) during the 2018/2019 and 2019/2020 seasons.

Soil Attribute	Season	
	2018/2019	2019/2020
Soil texture	Clay loam	Clay loam
Organic matter (%)	1.51	1.48
Electric conductivity (EC; dS m^−1^)	11.14	12.09
Field capacity (%)	29.4	31.1
pH	8.35	8.58
Soil bulk density (g cm^−3^)	1.42	1.38
Total porosity (%)	45.25	47.65
Cations (meq L^−1^)		
Na^+^	17.78	18.65
K^+^	10.73	11.86
Mg^+2^	14.76	16.23
Ca^+2^	16.54	18.29
Anions (meq L^−1^)		
Cl^−^	23.56	24.21
HCO_3_^−^	17.22	19.67
SO_4_^−2^	19.03	21.15

**Table 2:** Meteorological data of the experimental sites during 2018/2019 and 2019/2020 growing seasons.

Year Month	2018/2019				2019/2020			
	Temperature (°C)		Rainfall (mm)	Relative Humidity (%)	Temperature (°C)		Rainfall (mm)	Relative Humidity (%)
	Max	Min			Max	Min		
Oct.	26.3	17.2	0.98	32.6	25.3	16.2	0.94	31.6
Dec.	25.9	15.3	0.85	34.2	24.9	14.3	0.82	33.2
Jan.	24.5	13.2	1.1	35.1	23.2	12.4	0.54	32.7
Feb.	22.3	10.3	3.1	46.2	20.3	11.1	3.32	42.4
Mar.	21.4	9.7	6.4	44.3	20.6	10.7	6.85	43.1
April	23.7	13.8	0.5	43.8	22.5	12.5	0.63	44.8

## Data Availability

The data that supports the findings of this study are contained within the article and available from the corresponding author upon reasonable request.
